# Cell–cell adhesion interface: orthogonal and parallel forces from contraction, protrusion, and retraction

**DOI:** 10.12688/f1000research.15860.1

**Published:** 2018-09-25

**Authors:** Vivian W. Tang

**Affiliations:** 1 Department of Cell and Developmental Biology, University of Illinois, Urbana-Champaign, IL, 61801, USA

**Keywords:** cell-cell, adhesion, force, actin, intercellular, lateral membrane, epithelial, contraction, protrusion, retraction

## Abstract

The epithelial lateral membrane plays a central role in the integration of intercellular signals and, by doing so, is a principal determinant in the emerging properties of epithelial tissues. Mechanical force, when applied to the lateral cell–cell interface, can modulate the strength of adhesion and influence intercellular dynamics. Yet the relationship between mechanical force and epithelial cell behavior is complex and not completely understood. This commentary aims to provide an investigative look at the usage of cellular forces at the epithelial cell–cell adhesion interface.

## Introduction

Mechanical force, which is neither genetically encoded nor biochemically tractable, is being recognized as a significant determinant in the behaviors and functions of cells and tissues ^1– 9^. Whereas biochemical information is encoded in parameters such as post-translational modifications, biophysical information is encoded in parameters such as magnitude, frequency, and duration. Yet how these biophysical parameters of mechanical force contribute to the regulation of molecular and cellular events is not understood. Despite great efforts from biologists, physicists, and engineers to probe mechanical forces in living cells and tissues ^10– 22^, it remains tremendously difficult to measure the biophysical variables of mechanical forces inside cells. The purpose of this commentary is to explore, in the context of cell–cell interactions, the properties and usages of mechanical forces that can support cellular and molecular work ^23– 26^.

At the epithelial cell–cell adhesion interface, mechanical force is powered predominantly by actin dynamics and their associated molecular motors, including (a) protrusive force driven by actin polymerization and myosin activities ^24, 27– 35^; (b) retraction force contributed by cortical actin depolymerization, plasma membrane tension, retrograde actin flow, and myosin activities ^36– 42^; and (c) contraction force provided by actin dynamics and cross-linked actomyosin II activities ^43– 48^. This commentary will not discuss individual adhesion molecules and cytoskeletal regulators, which had been recently reviewed ^49, 50^.

### The three-dimensional epithelial cell

The epithelial cell forms distinct membrane domains by using two sets of adhesion molecules: one set for adhesion to extracellular matrix and another set for adhesion between cells. By having two sets of adhesion systems, an epithelial cell establishes structural and spatial organization with (a) a lateral membrane organized by cell–cell adhesions, (b) a basal membrane organized by cell–matrix adhesions, and (c) a free surface at the apical membrane. Through interactions with its environment and neighbors, the epithelial cell acquires emergent properties integral to the functions of epithelial tissues, such as coordinated multi-cellular junctional constriction and collective movement ^51– 59^.

**Figure 1.**
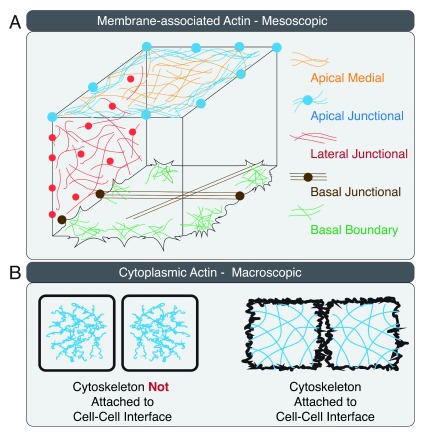
Actin organization in a three-dimensional epithelial cell. ( **A**) Actin arrangement on the apical, lateral, and basal membranes of the epithelial cell is illustrated at the mesoscopic cellular level. ( **B**) Connectivity between cell–cell interface and the actin cytoskeleton is illustrated at the macroscopic multi-cellular level.

The three membrane domains of the epithelial cell contain actin nucleation factors, actin cross-linking proteins, and myosin II filaments that create distinct actomyosin II networks ( Figure 1A) ^25, 60– 62^. The lateral membrane is further organized into apical, lateral, and basal intercellular interactions with distinct protein compositions, actin dynamics, and contractile properties ^63^. Mechanical forces generated at the apical, lateral, and basal actomyosin II cytoskeleton are transmitted to cell–cell contacts via linkage to adhesion proteins or the plasma membrane ( Figure 1B).

### Direction of forces

**Figure 2.**
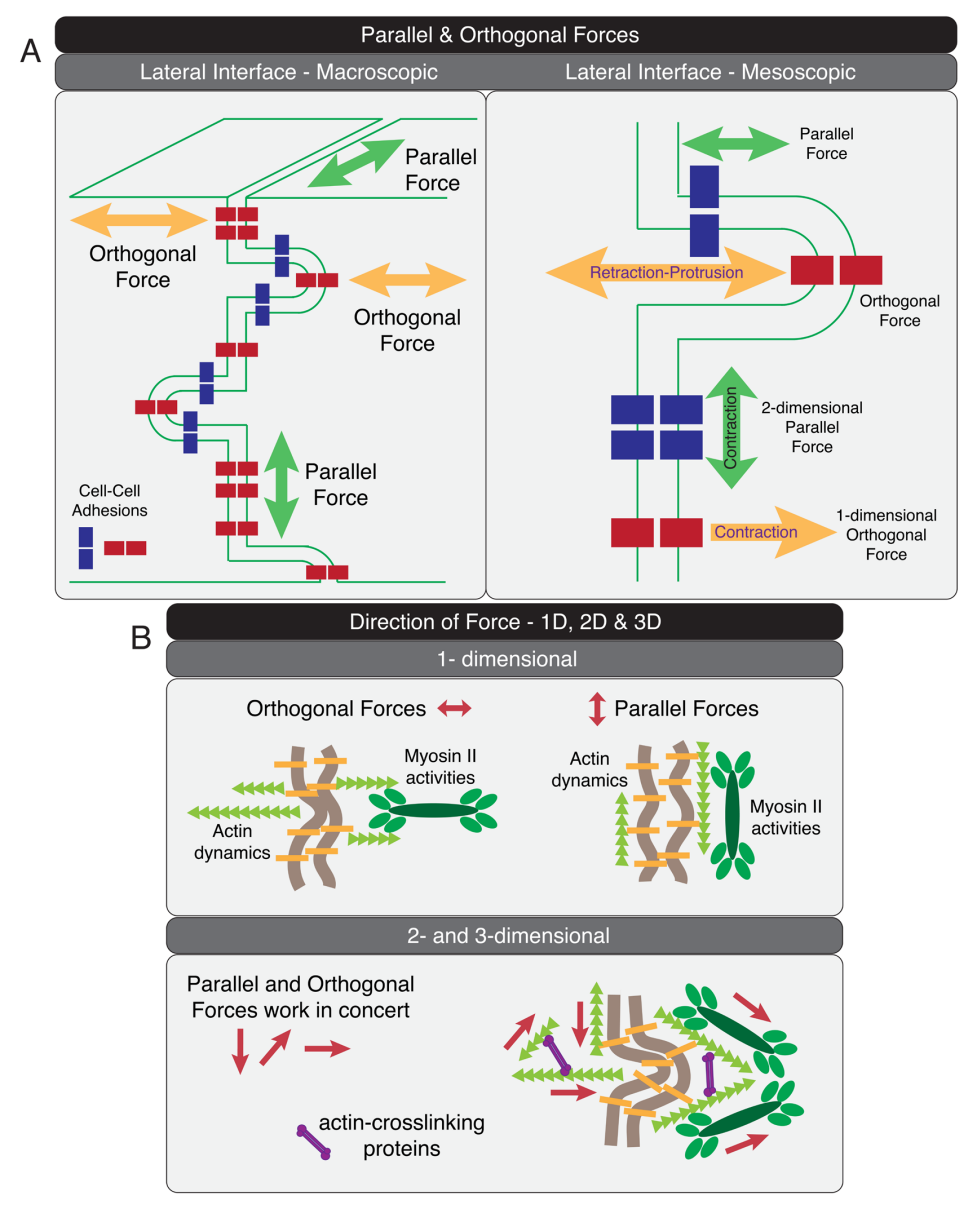
Direction of forces at the lateral cell–cell adhesion interface. ( **A**) Parallel and orthogonal forces are shown at the macroscopic multi-cellular (left panel) and mesoscopic cellular (right panel) scales. Cell–cell adhesions at the tip of a lateral membrane protrusion experience orthogonal force, whereas cell–cell adhesions at the trunk of the same membrane protrusion experience parallel force (right panel). ( **B**) Forces in one, two, and three dimensions can be generated through spatial organization of actin dynamics and actomyosin activities. Orthogonal and parallel one-dimensional forces can be generated with actin filaments arranged orthogonal and parallel to the plasma membrane, respectively (upper panel). Orthogonal and parallel two- and three-dimensional forces can be generated with a cross-linked actin network to support processes on the lateral cell–cell adhesion interface (lower panel).

Unlike chemical signaling that is driven by random walks and does not have intrinsic directionality, mechanical force intrinsically has a direction that is important to guide biological processes ^64– 68^. The directionality of the mechanical force is heavily influenced by the spatial organization of the actomyosin II cytoskeleton. Mechanical forces on the lateral adhesion interface are frequently orthogonal or parallel to the cell boundary ( Figure 2A, left panel). One exception to this generalization is at folded membranes ( Figure 2A, right panel). When protrusions and retractions are formed on the lateral membrane, adhesion proteins are no longer in the same orientation as the cell–cell boundary. Consequently, the direction of force exerted on adhesion molecules would be dependent on their relative positions. Cell–cell adhesions formed at the tip of an intercellular membrane protrusion would experience orthogonal protrusion–retraction force, whereas cell–cell adhesions found at the trunk of an intercellular membrane protrusion would experience parallel drag force on the plane of the plasma membrane ( Figure 2A, right panel).

Mechanical force applied in a directional manner could elicit different molecular and cellular events on the cell–cell adhesion interface ^65, 66, 69^. At the microscopic scale ( Figure 2B, upper panel), actin dynamics can create one-dimensional (1D) protrusion and retraction forces orthogonal to the lateral membrane. Anti-parallel myosin II filaments on linear actin filaments can create 1D contraction force. Actin filaments arranged parallel to the membrane would support force generation parallel to the cell–cell interface. Organization of branched actin networks using actin cross-linking proteins would create parallel and orthogonal forces to drive 2D and 3D events ( Figure 2B, lower panel). Spatial and temporal control of forces in one, two, and three dimensions can support distinct force-dependent processes on the lateral membrane.

### Pushing and pulling forces to drive kinetic processes

**Figure 3.**
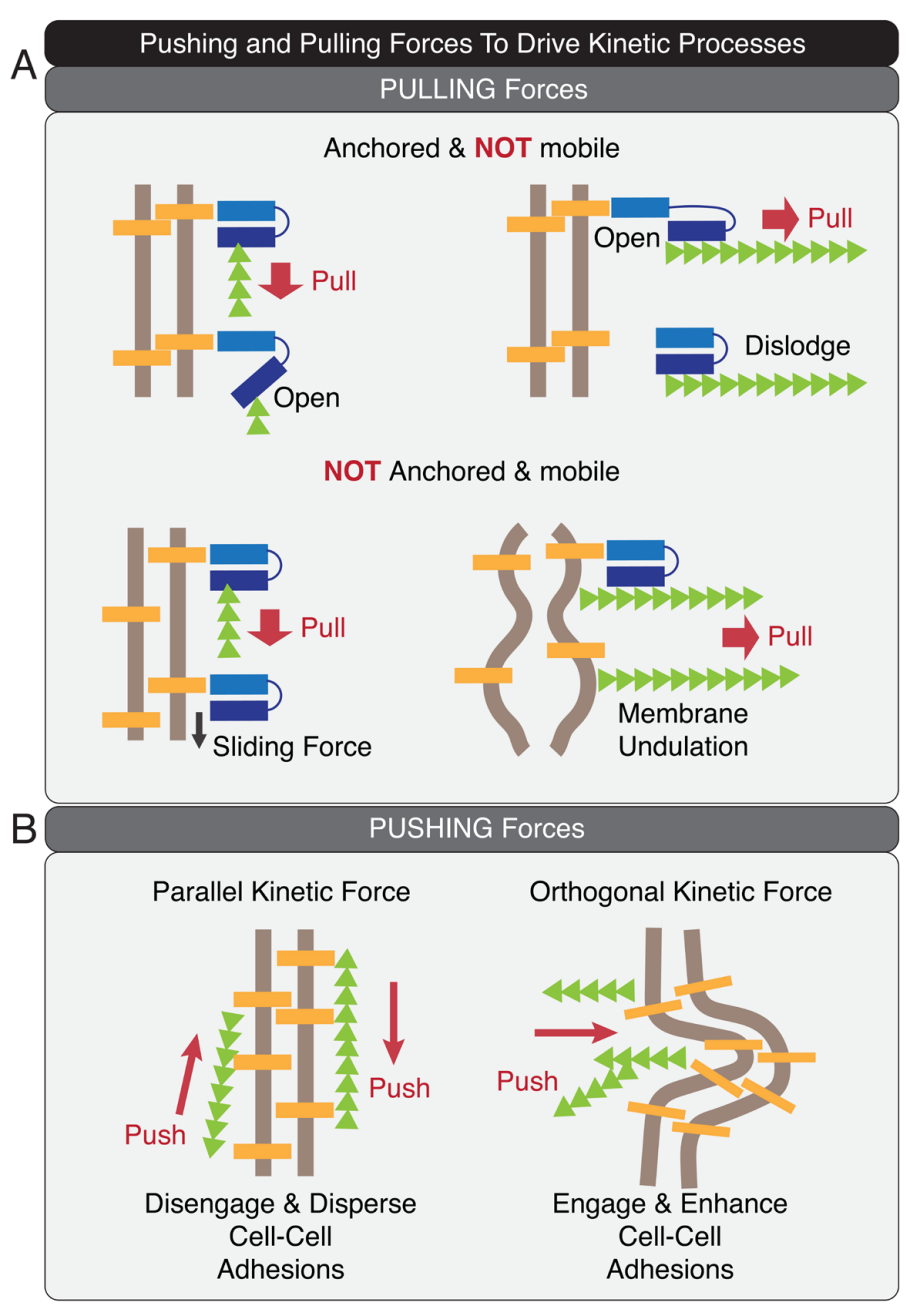
Pushing and pulling to drive kinetic processes. ( **A**) Pulling on adhesions and membrane can break intra-molecular and inter-molecular bonds, cause movement of adhesions on the membrane, and create undulations on the cell–cell adhesion interface. ( **B**) Pushing parallel to the lateral membrane can disengage and disperse adhesions, resulting in mixing of the membrane components. Pushing orthogonal to the lateral membrane can increase cell–cell contact area and time to enhance adhesion engagement.

The kinetic energy produced by mechanical forces can be used to create movement, resulting in productive work such as pushing and pulling ( Figure 3) ^41, 70– 76^.

Pulling forces can act on adhesions to unfold or disengage a protein by breaking intra-molecular or inter-molecular bonds, effectively changing the energy landscape of the cell–cell adhesion interface ( Figure 3A) ^77– 82^. Weaker interactions can be disrupted by weak forces but stronger interactions can resist weak forces and are selectively preserved. Thus, the input of different levels of kinetic energy would create different metastable states of the epithelial cell ^83^. For mobile and unanchored adhesions, pulling forces can result in sliding of the adhesion complex. Directional pushing can result in biased movement of the mobile fraction. Pulling orthogonally to the cell–cell interface can cause undulations of the lateral membrane, creating intercellular space and increasing the distance between apposing cell–cell adhesion molecules.

Pushing the lateral membrane can influence the distribution of protein complexes and membrane dynamics on the cell–cell adhesion interface ( Figure 3B) ^70, 75, 84– 89^. Pushing parallel to the membrane can disperse proteins and lipids, resulting in mixing of adhesion complexes and membrane components. By contrast, pushing orthogonal to the membrane can drive opposing cells together to increase contact time and area for adhesion engagement.

In summary, the spatial constraint created by organizing actin dynamics and actomyosin cytoskeleton can determine the magnitude, direction, and dimensionality of force. These biophysical properties of force are recognized by the 3D epithelial cells to drive different molecular and cellular events. Importantly, the orientation of the adhesion proteins on the plane of the plasma membrane determines the molecular outcome and ultimately the cellular effect of mechanical force on the cell–cell adhesion interface ^90– 92^.

### Pushing and pulling forces to drive thermodynamic processes

**Figure 4.**
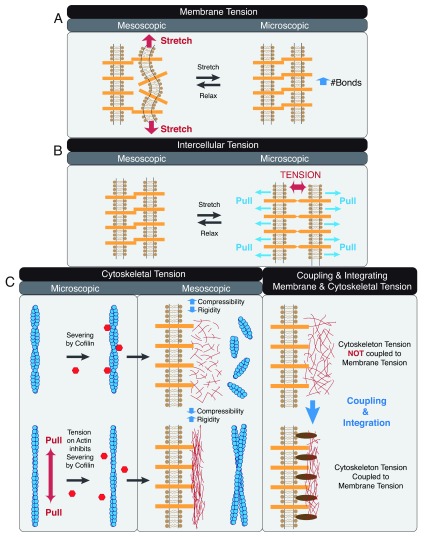
Pushing and pulling to create tension. ( **A**) Stretching the membrane increases membrane tension at the mesoscopic lateral membrane level and can enhance intercellular interactions at the microscopic molecular level. ( **B**) Pulling orthogonal to the membrane increases intercellular tension on the mesoscopic lateral membrane level and can enhance tension at cell–cell adhesion molecules at the microscopic molecular level. ( **C**) Pulling actin filament alters actin dynamics and decreases cofilin severing at the microscopic molecular level (left panel). Changing the length distribution and organization of actin filaments alters cytoskeletal tension, ultimately affecting the compressibility and rigidity of cortical actin associated with the lateral membrane at the mesoscopic lateral membrane level (middle panel). Coupling membrane and cytoskeleton tension to adhesion proteins allows integration of intercellular, membrane, cytoskeletal forces on the lateral cell–cell adhesion interface (right panel).

The energy supplied by mechanical forces can be stored as elastic energy to build up tension (a) on the plasma membrane ( Figure 4A) ^93– 96^, (b) on cell–cell adhesion molecules ( Figure 4B) ^97^, and (c) within the actin cytoskeleton ( Figure 4C) ^98– 101^. At the mesoscopic level, tension can be coupled and integrated to facilitate processes on the lateral interface ^56, 102, 103^.

Actin dynamics and actomyosin activities can increase membrane tension by stretching the membrane ( Figure 4A) ^104– 106^. Tension generated on the plane of the membrane will decrease undulation ^107– 109^, creating a taut region that supports lateral clustering of adhesion proteins ^103, 110– 112^. Orthogonal pulling force on the membrane can increase intercellular tension by stretching the extracellular domains of cell–cell adhesion proteins ( Figure 4B) ^113^.

Underneath the plasma membrane, tension can be generated in the cortical cytoskeleton ( Figure 4C) ^114, 115^. Pulling actin increases filament tension and decreases its susceptibility to severing by cofilin ( Figure 4C, left panel) ^116^ and results in changes in actin dynamics and filament stability ^117, 118^. Altering filament length and actin stability would affect connectivity of the actomyosin network and force transmission ^119– 122^. Pulling force on actin filaments could alter the spatial organization of the entire actomyosin network, resulting in mesoscopic differences in compressibility, stability, and rigidity ( Figure 4C, middle panel) ^123– 127^.

Plasma membrane and cytoskeletal tension can be coupled to create an integrated membrane–adhesion–cytoskeleton ensemble with properties that are more complex than the sum of the individual components ( Figure 4C, right panel) ^56, 91, 128– 137^. Thus, the energy input by pushing and pulling forces to generate tension on the lateral membrane, adhesion proteins, and cortical actin cytoskeleton ultimately results in the emergence of new properties and organization at the cell–cell adhesion interface ^12, 138– 141^.

### Magnitude of force

The reason to consider the magnitude of force is twofold. First, the magnitude of force dictates which process it can perform ( Table 1, upper panel) ^27, 40, 78, 142– 148^. Whether the mechanical force is meant to create tension, unfold protein, cause movement, or mix components on the membrane would depend on the energy required for the specific process. The magnitude of force can have a differential effect on proteins; weak force will break weaker bonds and preserve stronger bonds, whereas strong force will break both weak and strong bonds. For example, unfolding alpha-catenin would require less force than detaching an E-cadherin homophilic bond. Thus, the magnitude of force, along with the intrinsic transition states of the cell–cell adhesion system, dictates the kinetic and thermodynamic pathways for epithelial processes such as adhesion remodeling and strengthening.

**Table 1.** Magnitude of forces. ( **Upper**) Different processes require different levels of force. ( **Lower**) Different molecular mechanisms produce different levels of force.

Process	Force
Filopodium extension	< 3 pN
Unfolding vinculin binding site on α-catenin	5-10-15 pN
Unfolding intramolecular auto-inhibitory sites on ZO-1	5-20 pN
Detaching N-WASP from Arp2/3-F-actin complex	6-7 pN
Lamellipodia pushing force	10-20 pN
Breaking single E-cadherin-E-cadherin bond (without actin binding)	15-25 pN
Breaking single E-cadherin-E-cadherin bond	35-50 pN
Filopodium Retraction force (with actin and plasma membrane)	50-2000 pN
Machine	Force
Actin Polymerization	1 pN
Formin-driven Barbed-end Actin Polymerization	1-2 pN
Myosin I motor on actin	1-2 pN
Myosin VI motor on actin	2 pN
Myosin II single motor head on actin	1-6 pN
Myosin II minifilament on actin	20-50 pN
Myosin II minifilament on α-actinin-crosslinked actin bundle	100 pN
Myosin II minifilament on α-actinin-crosslinked actin network	1 micro N

Second, the magnitude of force dictates which mechanism can supply the force ( Table 1, lower panel) ^130, 149– 152^. Polymerization of a single actin filament supplies less force than a bundle of parallel actin filaments. To generate protrusion, a bundle of actin must polymerize in concert and work together ^153^. However, if force is needed only to push an adhesion protein closer to its binding partner on the membrane, a single actin filament may supply enough energy to promote biased diffusion. A myosin pulling on an actin filament produces less force than a bundle of myosin pulling on a bundle of parallel actin filaments. Anti-parallel myosin filaments pulling on parallel bundles of actin filaments produce less force than pulling on a cross-linked network of actin filaments. Thus, epithelial cells control the amount of force available by regulating the spatial distribution of actin dynamics and actomyosin II activities to dictate which molecular process is being executed at the lateral cell–cell adhesion interface ^154^.

## Conclusions

Mechanical force plays an important role at the cell–cell adhesion interface. How mechanical force regulates cellular and molecular processes depends on its encoded properties, including directionality and magnitude. Additional biophysical parameters such as frequency, duration, and waveform that are instructive signals for other biological regulatory systems such as calcium signaling and neuronal firing are also encoded in cellular mechanical forces, but their contribution to epithelial cell–cell interaction is currently unknown because of a lack of experimental data and approaches. However, if epithelial cells could encode and tune frequency, duration, and waveform in mechanical forces, it would greatly increase the parameter space, allowing epithelial cells to build complexity. Future development of techniques to measure cell-generated mechanical force will provide formal assessment of these biophysical variables in regulating epithelial cell–cell interactions and tissue behaviors.
